# A Pacific Culture among Wild Baboons: Its Emergence and Transmission

**DOI:** 10.1371/journal.pbio.0020106

**Published:** 2004-04-13

**Authors:** Robert M Sapolsky, Lisa J Share

**Affiliations:** **1**Department of Biological Sciences and Department of Neurology and Neurological Sciences, Stanford UniversityStanford, CaliforniaUnited States of America; **2**Institute of Primate Research, National Museums of Kenya KarenNairobiKenya

## Abstract

Reports exist of transmission of culture in nonhuman primates. We examine this in a troop of savanna baboons studied since 1978. During the mid-1980s, half of the males died from tuberculosis; because of circumstances of the outbreak, it was more aggressive males who died, leaving a cohort of atypically unaggressive survivors. A decade later, these behavioral patterns persisted. Males leave their natal troops at adolescence; by the mid-1990s, no males remained who had resided in the troop a decade before. Thus, critically, the troop's unique culture was being adopted by new males joining the troop. We describe (a) features of this culture in the behavior of males, including high rates of grooming and affiliation with females and a “relaxed” dominance hierarchy; (b) physiological measures suggesting less stress among low-ranking males; (c) models explaining transmission of this culture; and (d) data testing these models, centered around treatment of transfer males by resident females.

## Abstract 2

A unique less-aggressive suite of behaviors that affects the overall structure and social atmosphere of a wild baboon troop potentially represents an intergenerational transfer of social culture

## Introduction

A goal of primatology is to understand the enormous variability in primate social behavior. Early investigators examined interspecies differences, e.g., that pair-bonding is more common among arboreal than terrestrial primates (Crook and Gartlan 1966). Attention has also focused on geographical differences in behavior within species (Whiten et al. 1999). Often, such differences reflect environmental factors (e.g., a correlation between quantities of rainfall and foraging time) or, in theory, could reflect genetic drift. However, increasing evidence suggests that group-specific traits can also represent “traditions” or “cultures” (the latter term will be used, commensurate with the near consensus among primatologists that the term can be appropriately applied to nonhuman primates).

As traditionally applied to humans, such “culture” can be defined as behaviors shared by a population, but not necessarily other species members, that are independent of genetics or ecological factors and that persist past their originators (Kroeber and Kluckhohn 1966; Cavalli-Sforza 2000; de Waal 2000; de Waal 2001). Thus defined, transmission of culture occurs in apes (McGrew 1998; Whiten et al. 1999; van Schaik et al. 2003), monkeys (Kawai 1965; Cambefort 1981; Perry et al. 2003), cetaceans (Noad et al. 2000; Rendell and Whitehead 2001), and fish and birds (Laland and Reader 1999; Laland and Hoppitt 2003). As particularly striking examples, chimpanzees *(Pan troglodytes)* across Africa demonstrate variability in 39 behaviors related to tool use, grooming, and courtship (Whiten et al. 1999), and the excavation of near-millenium-old chimpanzee tools has been reported (Mercader et al. 2002).

Nearly all such cases of nonhuman culture involve either technology (for example, the use of hammers for nut cracking by chimpanzees), food acquisition, or communication. In this paper, we document the emergence of a unique culture in a troop of olive baboons *(Papio anubis)* related to the overall structure and social atmosphere of the troop. We also document physiological correlates of this troop atmosphere, the transmission of relevant behaviors past their originators, and possible mechanisms of transmission.

## Results/Discussion

### Circumstances Leading to the Emergence of a Unique Culture

In the early 1980s, Forest Troop slept in trees 1 km from a tourist lodge. During that period, an open garbage pit was greatly expanded at the lodge. This attracted an adjacent baboon troop, Garbage Dump Troop, which slept near the pit and foraged almost exclusively there.

By 1982, many Forest Troop males went to the garbage pit at dawn for food. While such refuse eaters did not differ in age distribution (data not shown) or average dominance rank from non–refuse eaters, they were more aggressive (Table 1); such aggressiveness could be viewed as a prerequisite in order to compete with Garbage Dump males for access to refuse. Refuse eaters were also involved in more dominance interactions within Forest Troop than were non–refuse eaters (note that frequency of dominance interactions is independent of outcome, and thus of rank).

**Table 1:**
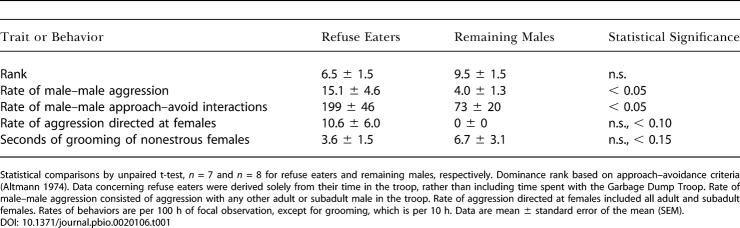
Characteristics of Forest Troop Males As a Function of Whether They Competed for Refuse with the Garbage Dump Troop

Statistical comparisons by unpaired t-test, *n* = 7 and *n* = 8 for refuse eaters and remaining males, respectively. Dominance rank based on approach–avoidance criteria (Altmann 1974). Data concerning refuse eaters were derived solely from their time in the troop, rather than including time spent with the Garbage Dump Troop. Rate of male–male aggression consisted of aggression with any other adult or subadult male in the troop. Rate of aggression directed at females included all adult and subadult females. Rates of behaviors are per 100 h of focal observation, except for grooming, which is per 10 h. Data are mean ± standard error of the mean (SEM)

In 1983, an outbreak of bovine tuberculosis occurred, originating from infected meat in the dump. From 1983 to 1986, most Garbage Dump animals died, as did all refuse-eating Forest Troop males (46% of adult males); no other Forest Troop animals died (Tarara et al. 1985; Sapolsky and Else 1987).

These deaths greatly altered Forest Troop composition, such that there were fewer adult males and more adult females; this more than doubled the female:male ratio (Table 2). By 1986, troop behavior had changed markedly, because only less aggressive males had survived.

**Table 2:**
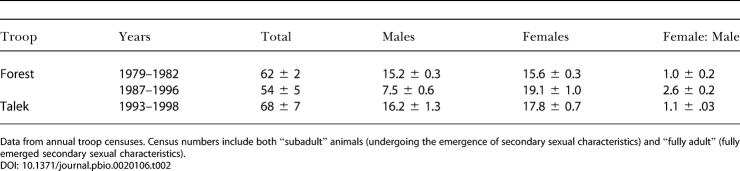
Troop Composition Before and After the Tuberculosis Outbreak

Data from annual troop censuses. Census numbers include both “subadult” animals (undergoing the emergence of secondary sexual characteristics) and “fully adult” (fully emerged secondary sexual characteristics)

Because of these events, observations of the troop were stopped, and only censusing was done until 1993. Research was begun on Talek Troop, approximately 50 km away.

In 1993, informal observation of Forest Troop indicated that the behavioral features seen by 1986 had persisted. Critically, by 1993, no adult males remained from 1983–1986; all current adult males had joined the troop following 1986. Thus, the distinctive behaviors that emerged during the mid-1980s because of the selective deaths were being carried out by the next cohort of adult males that had transferred into the troop. Focal sampling on Forest Troop recommenced in 1993, in order to document this phenomenon. Data from Forest Troop 1993–1996 (henceforth, F93–96) were compared with two other data sets that served as controls: observations from 1993–1998 on the Talek Troop (henceforth T93–98), and observations of Forest Troop itself prior to the deaths (1979, 1980, 1982; henceforth F79–82). These two control data sets did not differ significantly from each other and were combined, henceforth T93–98/F79–82.

### Atypical Features of the Behavior of Forest Troop Males

#### Male–male dominance interactions

Males of F93–96 and T93–98/F79–82 had similar rates of approach–avoidance dominance interactions (data not shown). Moreover, dominance stability did not differ, as measured by the percentage of approach–avoidance interactions which represented a reversal of the direction of dominance within a dyad of males of adjacent rank (16% ± 5% and 20% ± 5% for F93–96 and T93–98/F79–82, respectively, n.s.). There was also no difference in the average tenure length of the highest-ranking male (approximately a year).

**Figure 1:**
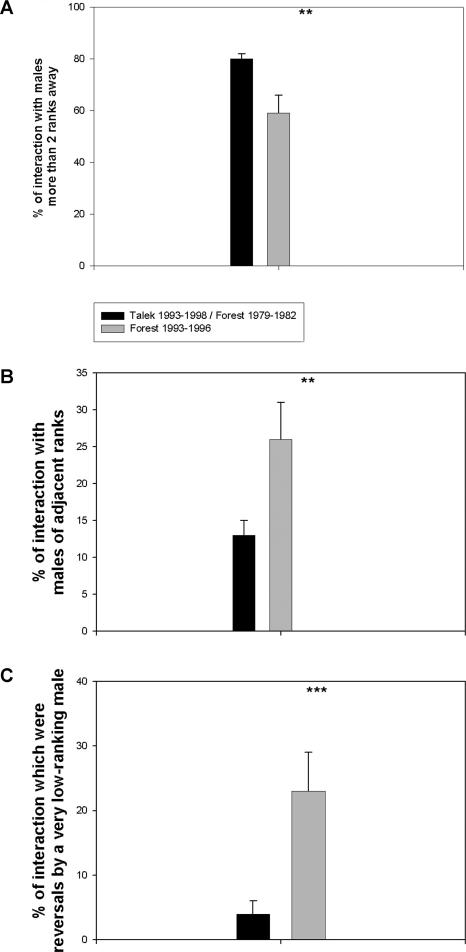
Quality of Male–Male Dominance Interactions (A) Percentage of male approach–avoidance dominance interactions occurring between males more than two ranks apart. (B) Percentage of male approach–avoidance interactions occurring between males of adjacent ranks. (C) Percentage of approach–avoidance interactions representing a reversal of the direction of dominance within a dyad by a male more than two steps lower ranking. Mean ± SEM, ** and *** indicate *p* < 0.02 and *p* < 0.01, respectively, by t-test, treating each male/year as a data point. Data were derived from a total of ten different males in F93–96, 31 different males in T93–98, and 19 different males in F79–82. Potentially, the result in (B) could have arisen from different numbers of males in F93–96 versus the other two troops (a smaller group size does not change the number of adjacent animals available to any given subject, but decreases the number of nonadjacent animals available). However, the same results were found if the numbers of males in the three troops were artificially made equal by excluding excess males from either the top or the bottom of the hierarchy (data not shown).

Despite those similarities, dominance behavior in F93–96 differed from the two control cases in ways that, arguably, made for less stress for low-ranking males. A first example concerns approach–avoidance dominance interactions between males more than two ranks apart in the hierarchy. The overwhelming majority of such interactions were won by the higher-ranking individual. Because a male is rarely seriously threatened by an individual more than two ranks lower in the hierarchy, interactions between individuals that far apart typically represent harassment of or displacement of the subordinate by the higher-ranking male, rather than true competition. In T93–98 and F79–82, approximately 80% of approach–avoidance interactions were between males more than two ranks apart in the hierarchy. In contrast, a significantly smaller percentage of approach–avoidance interactions were soin F93–96 (Figure 1A). Instead, a disproportionate percentage of F93–96 dominance interactions occurred among males of adjacent ranks (with, as noted, no difference in dominance stability)(Figure 1B). Moreover, high-ranking males in F93–96 were more “tolerant” of very low-ranking males, as there was a disproportionately high number of reversals with males more than two steps lower in the hierarchy (Figure 1C). Thus, in F93–96, with a typical level of dominance stability, approach–avoidance dominance interactions were concentrated among closely ranking animals, with low-ranking males being more tolerated and less subject to harassment and/or displacement by high-ranking males.

#### Aggression

Patterns of aggression also differed between F93–96 and T93–98/F79–82 in a way that suggested a less stressful environment for subordinates in F93–96. The troops had similar overall rates of aggressive interactions (Table 3). However, aggression in F93–96 was more likely than in the control troops to occur between closely ranked animals (i.e., within two rank steps), rather than to reflect high-ranking males directing aggression at extremely low-ranking ones; the latter type of interaction is particularly stressful for a subordinate, because of its typical unpredictability. Moreover, F93–96 males were less likely than T93–98/F79–82 males to direct aggression at females.

**Table 3:**
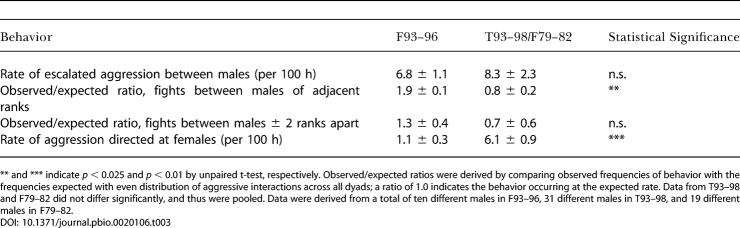
Patterns of Aggression in Forest Troop 1993–1996 versus Talek Troop 1993–1998 and Forest Troop 1979–1982

** and *** indicate *p* < 0.025 and *p <* 0.01 by unpaired t-test, respectively. Observed/expected ratios were derived by comparing observed frequencies of behavior with the frequencies expected with even distribution of aggressive interactions across all dyads; a ratio of 1.0 indicates the behavior occurring at the expected rate. Data from T93–98 and F79–82 did not differ significantly, and thus were pooled. Data were derived from a total of ten different males in F93–96, 31 different males in T93–98, and 19 different males in F79–82

We examined the data for reconciliative behavior (i.e., affiliative behaviors between pairs following aggressive interactions [de Waal and van Roosmalen 1979]) in F93–96 and T93–98/F79–82. However, we saw no male–male reconciliation in any troop, in agreement with prior reports (Cheney et al. 1995).

#### Affiliative behaviors

**Figure 2:**
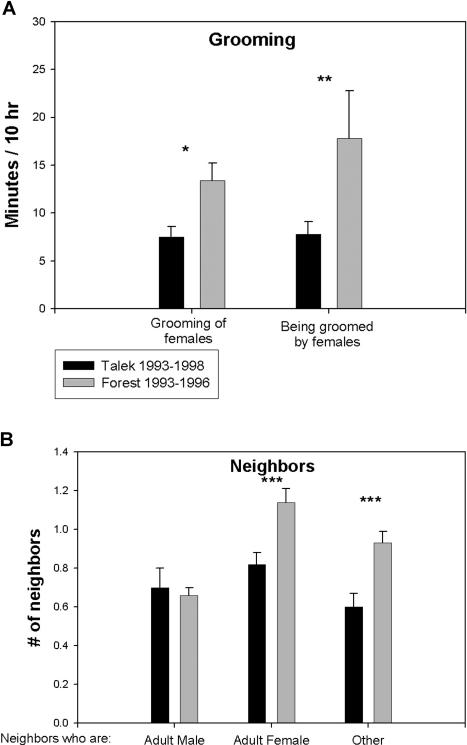
Quality of Affiliative Behaviors (A) Amount of grooming involving adult males in Forest Troop 1993–1996 and Talek Troop 1993–1996. The first pair of columns represents mean time adult males spent grooming adult females; the second pair, mean time adult males were groomed by adult females. (B) Comparison of average number of neighbors (i.e., within 3 m) of adult males in the two troops. Mean ± SEM. *, **, and *** indicate *p* < 0.05, 0.02, and 0.01, respectively, by unpaired t-test. Data were derived from a total of ten different males and 17 different females in F93–96, 31 different males and 21 different females in T93–98, and 19 different males and 23 different females in F79–82.

Quantitative data on affiliative behaviors were not available for F79–82. However, F93–96 males socially groomed more often than did control T93–98 males (Figure 2A) this difference was due to more grooming between males and females. F93–96 males were also in close proximity to other animals more often than were T93–98 males (Figure 2B). While males did not differ between troops in the average number of adult male neighbors (i.e., within 3 m), F93–96 males were more likely than T93–98 males to have adult females, infants, adolescents, and juveniles as neighbors.

#### Sexual behavior

Sexual behavior did not differ between F93–96 and T93–98/F79–82. The percentages of nonpregnant, nonlactating females in estrus per day did not differ (27% ± 7% and 30% ± 4%, respectively, n.s.). Moreover, the relationship between male rank and reproductive success did not differ (*R^2^* of correlation between rank and reproductive success: 0.25 ± 0.25 and 0.54 ± 0.10, respectively, n.s.).

### Physiological Correlates of Behavioral Features of Forest Troop

Thus, F93–96 males had high rates of affiliative behaviors, and low-ranking males were subject to low rates of aggressive attack and subordination by high-ranking males. In a stable hierarchy, low-ranking baboon males show physiological indications of being stressed, including elevated basal levels of glucocorticoids (the adrenal hormones secreted in response to stress), hypertension, and decreased levels of high density lipoprotein cholesterol, growth factors, and circulating lymphocytes (Sapolsky 1993; Sapolsky and Share 1994; Sapolsky and Spencer 1997). We tested whether subordinate males in F93–96 were spared the stress-related physiology of subordination seen in other troops.

**Figure 3:**
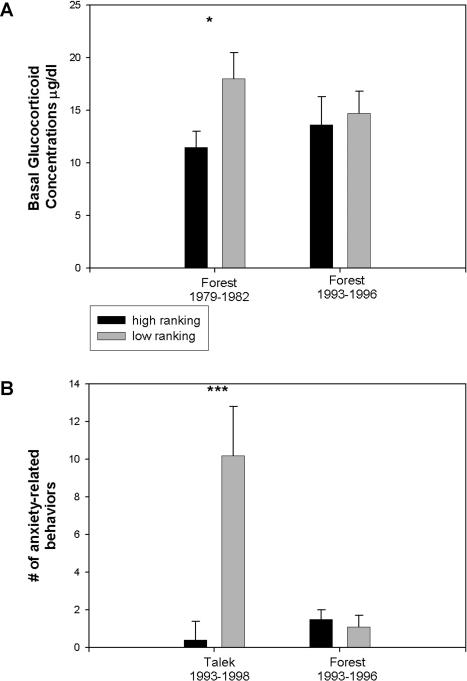
Stress-Related Physiological Profiles (A) Basal glucocorticoid levels (μg/100 ml). Males were split into higher- and lower-ranking 50%, by approach–avoidance criteria. The primate glucocorticoid, cortisol, was measured by radioimmunoassay. (B) Number of anxiety-related behaviors observed 10–20 min after β-carboline-3-carboxylic acid administration (M-156, Research Biochemicals International, Natick, Massachusetts, United States), after subtracting the number observed 10–20 min after vehicle administration (dextrin in 1 ml saline); 0.5 g of the drug in 1ml saline was delivered intramuscularly by dart syringe (Pneu-Dart, Inc., Williamsport, Pennsylvania, United States) fired from a blowgun at 5 m. Mean ± SEM. * and *** indicate *p* < 0.05 and *p* < 0.01, respectively, by unpaired t-test. Data were derived from a total of ten different males in F93–96, 31 different males in T93–98, and 18 different males in F79–82.

This was the case (Figure 3A). In F79–82, i.e., prior to the tuberculosis outbreak, subordination was associated with elevated basal levels of glucocorticoids, as in other species in which subordination entails extensive stressors and low rates of coping outlets (Sapolsky 2001). While glucocorticoids aid in surviving an acute physical stressor, chronic overexposure increases the risk of glucose intolerance, hypertension, ulcers, and reproductive and immune suppression (Sapolsky et al. 2000). In contrast to this picture in F79–82, in which subordination was associated with a physiology suggesting a chronic state of stress, subordinate F93–96 males did not have elevated basal glucocorticoid levels (levels were unavailable for T93–98).

Subordinate F93–96 males were spared another stress-related physiological marker. Experimental anxiety was induced by darting males, intramuscularly, with β-carboline-3-carboxylic acid, a benzodiazepine receptor antagonist which induces behavioral and physiological indices of anxiety in primates (benzodiazepine receptors bind tranquilizers such as valium and librium and mediate their anxiolytic effects)(Ninan et al. 1982). Males were darted on days when they had not had a fight, injury or mating. As a control, they were darted on separate days with vehicle alone (order of dartings randomized). Males were then monitored by an observer unaware of treatment.

β-carboline-3-carboxylic acid had no effect on behavior in high-ranking males in T93–98 or F93–96 (Figure 3B).The drug increased anxiety-related behaviors in low-ranking males in T93–98 but not in F93–96 (the recorded anxiety-related behaviors were self-scratching, rhythmic head shaking, assuming a vigilant stance, repeated wiping of nose, and jaw grinding in a solitary male [Ninan et al. 1982; Aureli and van Schaik 1991; Castles et al. 1999]).

Thus, in the more typical F79–82 and T93–98 troops, subordination had distinctive stress-related physiological correlates. In contrast, F93–96 males lacked these rank-related differences.

### Potential Mechanisms Underlying Transmission of This Culture

A decade after the deaths of the more aggressive males in the troop, Forest Troop preserved a distinct social milieu accompanied by distinct physiological correlates. Critically, as noted, no adult males in F93–96 had been troop members at the end of the tuberculosis outbreak. Instead, these males had subsequently transferred in as adolescents, adopting the local social style. A number of investigators have emphasized how a tolerant and gregarious social setting facilitates social transmission (e.g., van Schaik et al. 1999), exactly the conditions in F93–96.

The present case of social transmission is reminiscent of some prior cases. For example, juvenile rhesus monkeys *(Macaca mulatta)* housed with stumptail macaques *(M. artoides)* assume the latter's more conciliatory style (de Waal and Johanowicz 1993). Moreover, anubis baboons *(Papio anubis)* and hamadryas baboons *(P. hamadryas)* differ in social structure, and females of either species experimentally transferred into a group of the other adopt the novel social structure within hours (Kummer 1971).

Several models have been hypothesized to explain transmission of cultures (Whiten et al. 1999; de Waal 2001; Galef 1990). For clarity, it is useful to first consider their application to an established example of transmission of a “technology” before then applying them to the transmission of the social milieu of F93–96. An example of the former is the nut cracking with stone hammers by West African chimpanzees (Boesch and Boesch 1983; Boesch 2003), a trait transmitted transgenerationally.

In “instructional models” of chimpanzee tool use, young are actively taught hammer use. In the case of F93–96, instructional models would involve new transfer males being subject to socially rewarding interactions (e.g., grooming) or aversive ones (e.g., supplantation or attack) contingent upon their assimilating the troop tradition. In such models, a key question is who “instructs.” Much as with the term “culture” being used with respect to animal behavior, the use of the term “instruction” has also generated some controversy, with some preferring the concept of “active behavioral modification” by others bringing about the change. As a striking example of that, when young male cowbirds learn to produce their local song, they initially produce an undifferentiated repertoire of songs, and females react to the production of appropriate dialect with copulation solicitation displays, thus providing positive reinforcement and shaping those behaviors (Smith et al. 2000).

In “observational models” applied to chimpanzee tool use, young learn nut cracking by observing and copying adults. As applied to F93–96, transfer males would model behavior upon that of resident males.

In “facilitation models” of the chimpanzee example, proximity to adults and their hammers increases the likelihood of the young experimenting with hammers and deriving the skill themselves. As applied to the baboons, male F93–96 behaviors would be an implicit default state where, in the absence of the more typical rates of male aggression (either male–male or male–female), females broadly tend to become more affiliative, and in the context of more affiliative female behavior, transfer males broadly tend to become less aggressive. As perhaps a way of stating the same, the default state may emerge because of the atmosphere of a troop with a high female:male ratio (with less need for male competition for access to estrus females).

Finally, a “self-selection model” may apply to the baboons, in which particular kinds of males were more prone to transfer into such a troop (note that the fact that males transferred in from an array of surrounding troops rules out the possibility of an additional model, in which the culture was continued by genetic means).

We assessed these models by analyzing cases where adolescent males transferred on known dates and were observed for at least 2–6 mo afterward. Thus, we searched for behavioral patterns involving new transfer males that might differ between F93–96 (five such transfers) and T93–98/F79–82 (12 transfers).

Many interactions involving new transfer males did not differ (Table 4). Transfer males in F93–96, T93–98, and F79–82 all attacked and supplanted females from feeding or resting sites at equal rates. Moreover, despite the different dominance structure among resident F93–96 males, resident males in F93–96, T93–98, and F79–82 all treated new transfer males similarly. There were similar latencies until transfer males were first lunged at by residents, and transfer males were involved in dominance and aggressive interactions at similar rates in all three troops (note that because there were half as many resident males in F93–96 as in T93–98 or F79–82, the rate of such interactions within any given resident/transfer male dyad would differ). We examined instances where resident males acted aggressively towards transfer males, determining whether such behaviors were more prevalent during the 20 min after aggressive behavior by the transfer male than at other, randomly selected times (de Waal and Yoshihara 1983; de Waal and Johanowicz 1993). We found no evidence for such contingent behavior (data not shown).

**Table 4:**
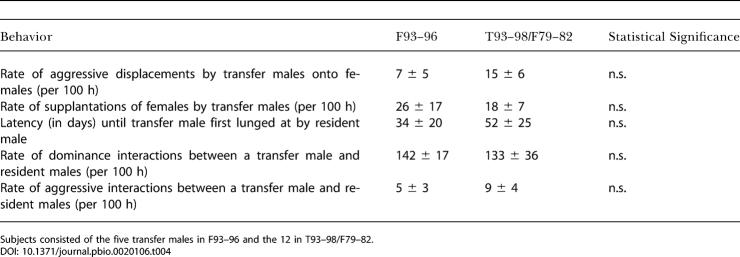
Behaviors of Newly Transferred Males

Subjects consisted of the five transfer males in F93–96 and the 12 in T93–98/F79–82

**Figure 4:**
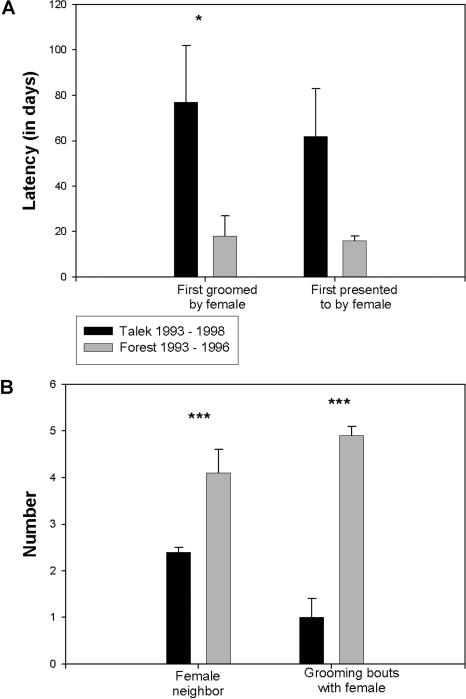
Quality of Interactions between Resident Females and Transfer Males (A) Latency, in days, until a newly transferred male is first groomed by a female (left) or presented to by a female (right). (B) Average number of adult female neighbors per scan (i.e., within 3 m; left) and average number of grooming bouts with females per 100 h of observation (right) for transfer males. Mean ± SEM. * and *** indicate *p* < 0.05 and *p* < 0.01, respectively, by unpaired t-test. Latency until first presented to by a female approached significance (*p* < 0.08). Data were derived from a total of ten different males and 17 different females in F93–96, and 31 different males and 21 different females in T93–98.

We then examined affiliative interactions between females and new transfer males, and found striking differences between F93–96 and T93–98/F79–82, in that F93–96 females treated new transfer males in the same affiliative manner that they treated resident males. F93–96 transfer males had a shorter latency until first being groomed by or presented to by a female than did T93–98/F79–82 transfer males (Figure 4A). (The differences between F93–96 and T93–98 did not arise from a single F93–96 female accounting for the much shorter latencies until presentation and grooming: three different females accounted for the first interactions with the five F93–96 transfer males). Moreover, F93–96 transfers sat in closer proximity to and had more grooming bouts with females than did T93–98/F79–82 transfers (Figure 4B). While estrous females are more likely than nonestrous females to interact with transfer males (Smuts 1999), the percentage of females in estrus did not differ among the troops (see above). In addition, F93–96 females did not seem to treat transfer males in a contingent manner (de Waal and Yoshihara 1983; de Waal and Johanowicz 1993). To test for this, we first examined instances where resident females were affiliative towards transfer males, determining whether this was more likely during the 20 min following an affiliative behavior on the part of the transfer male than at other, randomly selected times. Second, we determined whether females were less likely to be affiliative during the 20 min following an aggressive behavior on the part of a transfer male. We found no evidence for either pattern (data not shown).

These data allow some insight as to the mechanisms of social transmission in F93–96 (without remotely allowing an analysis fine-grained enough to see whether these mechanisms were equally relevant to the transmission of all the components of the F93–96 culture, namely the low rates of male aggression, the high rates of female affilitation, and the relaxed dominance structure). The lack of contingency in the treatment of transfer males by residents argues against instruction; commensurate with this, there is relatively little evidence for “instruction” in nonhuman primate cultural transmission (de Waal 2001; for an exception, see Boesch 1991). The similar rates of displacement behaviors by transfer males onto females in all three troops argue against self-selection (i.e., the possibility that F93–96 transfer males already behaved differently than transfer males elsewhere). This is not surprising. While adolescent male baboons may transfer repeatedly before choosing a troop (Pusey and Packer 1986), as well as later in life (Sapolsky 1996), we have seen little evidence among these animals of the systematic sampling of different troops required by a self-selection model.

The data instead support either observational or facilitative/default models. Insofar as resident males in all troops interacted with transfer males similarly, transmission in F93–96 could have involved observation only if such observations were of how resident males interacted with females or each other. Some, but not all, studies support observational models of social transmission in other primates (Visalberghi and Fragaszy 1990; Whiten 1998; Boesch 2003; Whiten et al. 2003); there are few data at present from baboons concerning this issue. As shown, F93–96 transfer males were had high rates of affilitative interactions with females. The preponderance of females in F93–96 is a plausible explanation for their unconditional (or, at least, less conditional) increase in tolerance of and affiliation with males (including transfer males), insofar as males in the troop had less numeric means to be aggressive to females. (Note that this skewed sex ratio continues in this troop to the present, for unknown reasons.) Thus, affilative data support a facilitative/default model only if it involves preferential sensitivity to the quality of interactions with females.

This analysis raises the possibility that there is no social transmission, but that the F93–96 pattern is merely the emergent outcome of the 2:1 female:male ratio. To test this, we analyzed the five available studies of baboon troops with adult female:male ratios of 2 or more which contained quantitative data comparable to the present data (Seyfarth 1976, 1978; Strum 1982; Bercovitch 1985; Noe 1994). The key question was whether those prior data more closely resembled those of F93–96 or the control troops. Previous data more closely resembled, and did not differ significantly from, data from the control troops for the percentage of time males groomed females (based on Seyfarth 1978), the percentage of time females groomed males (Seyfarth 1978), the rate of intersexual aggression (Seyfarth 1976, 1978), the structure of male–male dominance (Noe 1995), or the structure of of male–male aggression (Strum 1982; Bercovitch 1985). In contrast, no quantitative measures more closely resembled F93–96. This strongly suggests that the F93–96 pattern is unique and is being uniquely maintained, rather than being the social structure that automatically emerges whenever a female-skewed female:male ratio occurs. Thus, insofar as a facilitative/default model is operating in this troop, it cannot be a relative paucity of males which “activates” a default state; instead, it would likely be the paucity of aggressive males.

The unconditional (or less conditional) nature of the default model is puzzling, in that it requires that females be relatively affiliative to recent transfer males who, nonetheless, are initially aggressive to them. This seems counter to the long-standing emphasis in primatology on individual relations (i.e., females are unlikely to be unable to distinguish between relatively unaggressive resident males and relatively aggressive newly transferred males). Precedent for this unexpected implication comes from the social epidemiology literature concerning “social capital,” in which health and life expectancy increase in a community as a function of communitywide attributes that transcend the level of the individual or individual social networks (Kawachi et al 1997).

In summary, we have observed circumstances that produced a distinctive set of behaviors and physiological correlates in a troop of wild baboons. Moreover, these behaviors were taken on by new troop members; while obviously not conclusive, the data suggest that this most likely occurs through observational or facilitative/default models. Finally, somewhat uniquely in nonhuman primate studies, these findings concern the intergenerational transfer of social, rather than material culture.

These findings raise some issues. There appear to be adverse health consequences of the stress-related physiological profile of subordination in typical baboon troops (Sapolsky 1993; Sapolsky and Share 1994; Sapolsky and Spencer 1997). The distinctive rank-related patterns of physiology in F93–96 suggest that subordinate males in that troop may be spared those pathologies. Another issue concerns the consequences of the culture of F93–96 remaining stable over some time. A hallmark of human culture is that it is cumulative (i.e., innovations are built upon each other), and there is only scant evidence, at best, for the same in nonhuman primates (Boesch 2003). It would thus be interesting to see if additional features of the F93–96 social tradition emerge with time.

A converse issue concerns circumstances that might destroy the F93–96 culture. The culture might be destroyed if numerous males transfer into the troop simultaneously, or if a male transfers in who, rather than assuming the F93–96 culture, instead takes advantage of it. Game theory suggests that F93–96 would be vulnerable to such “cheating.” Another issue concerns the fate of natal males from F93–96 when they transfer elsewhere. Reciprocal altruism models (Axelrod and Hamilton, 1981) suggest that if one F93–96 male transfers elsewhere and continues his natal behavioral style, he will be at a competitive disadvantage. However, should two F93–96 males simultaneously join another troop and maintain F93–96–typical interactions between them, they might be at a competitive advantage. This might represent a means to transmit this social style between troops.

Finally, these findings raise the issue of their applicability to understanding human social behavior and its transmission. Human history is filled with examples of the selective loss of demographic subsets of societies (e.g., the relative paucity of adult men following the American Civil War or the relative paucity of girls in contemporary China due to male-biased reproductive technology practices and female-biased infanticide). The present data suggest that demographic skews may have long-term, even multigenerational consequences, including significant changes in the quality of life in a social group.

## Materials and Methods

Subjects were a troop, Forest Troop, of olive baboons *(Papio anubis)* living in the Masai Mara Reserve of Kenya. Olive baboons live in multimale troops of 30–150 animals, with polygamy and considerable male–male aggression. Males change troops at puberty and, as adults, achieve ranks in somewhat fluid dominance hierarchies. In contrast, females remain in their natal troop, inheriting a rank one below that of their mother.

Subjects were observed each summer from 1978–1986, and continuously since 1993. An additional troop, Talek Troop, was observed continuously since 1984. Behavioral data were collected as 20-min focal samples (Altmann 1974). During years of only summer observation (Forest Troop, 1978–1986), 45 samples were collected per subject per season; otherwise, an average of three samples per subject per week were collected throughout the year. Sampling was distributed throughout the day in the same fashion for each individual. During samples, social behavior, feeding, and grooming were recorded. Rankings were derived from approach–avoidance interactions, which included avoidances, supplants, and presentations, in the absence of aggression. Escalated aggression included open-mouthed lunges, chases, and bites. Nearest neighbor scans were done before and after each sample.

Reproductive success was indirectly estimated from frequencies of matings and consortships (maintenance of exclusive mating with and proximity to an estrous female for at least one sample). The value of any given consortship or mating was adjusted by the probability of a fertile mating occurring that day (Hendrickx and Kraemer 1969).

Endocrine data were collected under circumstances allowing for measures of basal steroid hormone levels (Sapolsky and Share 1997). Subjects were darted unaware with anesthetic from a blowgun syringe between 7 A.M. and 10 A.M., and only on days on which they were not sick, injured, in a consortship, or had not recently had a fight. Blood samples were collected within 3 min of anesthetization.

## Abbreviations

SEM: standard error of the mean
